# The Silent Threat: Unraveling the Impact of Rabies in Herbivores in Brazil

**DOI:** 10.3390/ani14162305

**Published:** 2024-08-08

**Authors:** Marcelo Cardoso da Silva Ventura, Jéssica Milena Moura Neves, Randyson da Silva Pinheiro, Marcos Vinicius Costa Santos, Elba Regina Sampaio de Lemos, Marco Aurelio Pereira Horta

**Affiliations:** 1 Federal Institute of Education of Piauí, Teresina 64000-040, Brazil; marceloventura@ifpi.edu.br; 2 Biosafety Level 3 Facility, Oswaldo Cruz Institute, Fiocruz, Rio de Janeiro 21040-900, Brazil; marco.horta@fiocruz.br; 3 Laboratory of Molecular Biology and Epidemiology, Federal Institute of Education of Piauí, Teresina 64000-040, Brazil; randysonpinheiro22@hotmail.com (R.d.S.P.); viniciusmarcos1212@gmail.com (M.V.C.S.); 4 Hantavirosis and Rickettsiosis Laboratory, Oswaldo Cruz Institute, Fiocruz, Rio de Janeiro 21040-900, Brazil; elemos@ioc.fiocruz.br

**Keywords:** bovine rabies, zoonotic disease, vaccination strategies, Brazil

## Abstract

**Simple Summary:**

Rabies, a deadly viral disease transmitted mainly through the saliva of infected animals like bats, dogs, and other wildlife, poses significant risks to both livestock and human health, particularly in Brazil. The virus predominantly spreads to herbivores, such as cattle, horses, and goats, through bites from vampire bats. Despite efforts to control the disease through vaccination programs, recent cases in Brazil highlight ongoing challenges. These include sporadic vaccination failures, the presence of vampire bats in some regions, and deforestation, which affects bat habitats and increases disease spread. The economic impact on Brazil’s livestock sector is considerable, resulting in losses in meat and milk production and posing public health risks. Effective management strategies involve not only maintaining robust vaccination schedules but also addressing environmental factors that facilitate rabies transmission. Increasing awareness and improving control measures are crucial to reducing the disease’s impact. By integrating vaccination efforts with habitat management and ongoing surveillance, Brazil can better protect its livestock and prevent the spread of rabies to humans, ensuring both animal welfare and public health.

**Abstract:**

Rabies, a zoonotic viral disease, poses a significant threat due to its adaptability to diverse environments. Herbivore rabies, predominantly affecting cattle, horses, and goats in Brazil, remains a concern, results in substantial losses in the livestock industry, and poses risks to public health. Rabies virus transmission, primarily through hematophagous bats in Latin America, underscores the need for effective strategies, and vaccination plays a crucial role in controlling herbivorous rabies, with systematic vaccination beingly the primary method. Efforts to control rabies in herbivores include vaccination campaigns, public awareness programs, and the enhancement of surveillance systems. Despite these initiatives, rabies persists and imposes an economic burden and a significant health risk. Economic impacts include losses in the livestock industry, trade restrictions on livestock products, and financial burdens on governments and farmers owing to control measures. Despite the considerable costs of campaigns, surveillance, and control, investing in rabies vaccination and control not only safeguards livestock, but also preserves public health, reduces human cases, and strengthens the sustainability of the livestock industry. Mitigating the impact of herbivorous rabies in Brazil requires integrated approaches and continuous investments in vaccination, surveillance, and control measures to protect public health and ensure the sustainability of the livestock industry, thus contributing to food and economic security.

## 1. Introduction

Rabies, a viral pathology that originated from a virus belonging to the genus *Lyssavirus*, is a zoonotic disease that has a notable impact on mammals, including humans, and is nearly invariably fatal upon the onset of symptoms [1]. Transmission of the rabies virus predominantly occurs through the saliva of infected animals, such as bats, dogs, raccoons, skunks, and foxes, via bites or scratches [2]. Although human-to-human transmission is exceptionally rare, it has been documented in cases of organ transplantation and, theoretically, through bites and saliva contact. The clinical outcomes of rabies are inevitable after the appearance of symptoms [3]. Immediate medical intervention, including post-exposure prophylaxis (PEP) with rabies vaccines and immunoglobulins, is effective in preventing the development of symptoms if administered promptly after exposure [4]. Although human rabies cases are relatively rare in developed nations with effective vaccination programs, the disease still poses significant concern in regions with limited access to healthcare and preventive measures [5].

Because of its ability to develop in various ecological niches, rabies poses a threat to both animals and humans, which highlights the importance of controlling its transmission in livestock populations. Rabies is a severe viral disease that predominantly affects herbivorous animals, such as cattle, horses, and goats [6]. In Brazil, it is a constant concern for the livestock industry, as the disease results in substantial losses in meat and milk production, in addition to posing a risk to public health, given that the infection can be transmitted to humans through contact with diseased animals [7]. Symptoms often observed in infected animals include aggressiveness, excessive salivation, dysphagia, and paralysis [8]. The transmission of the rabies virus to herbivores predominantly occurs through bites from hematophagous bats, notably vampire bats, which are the main reservoirs of the virus in rural environments. Although rabies control in herbivores in Brazil is largely achieved through the systematic vaccination of animals, particularly cattle and horses, the sporadic occurrence of cases suggests persistent challenges [9,10]. Recent disease reports may be attributed to various factors, including vaccination failures, the presence of hematophagous bats in certain regions, and the introduction of the virus into previously disease-free areas, which often results from ecological disturbances, such as deforestation [11].

The economic losses in the livestock industry in Brazil from herbivore rabies, predominantly affecting cattle, horses, and goats, pose a serious regional problem. The links between substantial landscape modification, particularly in the Amazon region of Brazil, show a direct link between environmental changes and disease emergence and the spread of rabies into new areas. This trend indicates the need for ‘One Health’ thinking to find solutions to this problem. One Health interventions are crucial in addressing the interconnected health of people, animals, and the environment, as highlighted by studies on the impact of landscape changes on disease dynamics [12].

It is crucial for veterinary health authorities and rural producers to maintain constant surveillance of the prevalence of rabies in herbivores, promote the regular vaccination of animals, and implement control measures aimed at hematophagous bats, such as the use of physical barriers and capture strategies [13]. In addition, education and awareness among those involved in the livestock industry are critical for mitigating the risk of disease transmission and ensuring the safety of both animals and people. Despite posing a substantial threat, the adoption of adequate prevention and control measures can minimize the impact of rabies on herbivores in Brazil.

### 1.1. Vaccination

In Brazil, strategies for rabies control in herbivores are diverse and encompass essential practices for mitigating the disease spread. Bovine vaccination plays a crucial role in livestock management by preventing the propagation of rabies, safeguarding animal health, and reducing the risk of transmission to humans and other animals [14]. This practice is fundamental to responsible cattle management and contributes to the overall well-being of animals and dependent communities. Bovine vaccination primarily relies on inactivated vaccines developed to combat rabies, which induce a robust immune response and stimulate antibody production [15]. Protocols often involve initial doses followed by periodic boosters tailored to the local context. Schedule planning considers factors such as the local prevalence of the disease, type of vaccine, and regional regulations to ensure protection against rabies and promote health security within the herd [16].

Regarding vaccination costs, both the Brazilian government and cattle producers have invested significantly in campaigns, surveillance, and rabies control. These include expenses for vaccines, veterinary supplies, logistics, training, supervision, and monitoring in rural and urban areas [17]. Producers also face additional costs for biosecurity measures and the hiring of specialized labor. Despite financial challenges, it is essential to recognize that investing in rabies vaccination and control not only protects cattle, but also preserves public health, reduces human cases, and strengthens the sustainability of the livestock industry, this contributing to the country’s food and economic security [18].

### 1.2. Herbivore Rabies

**Figure 1:**
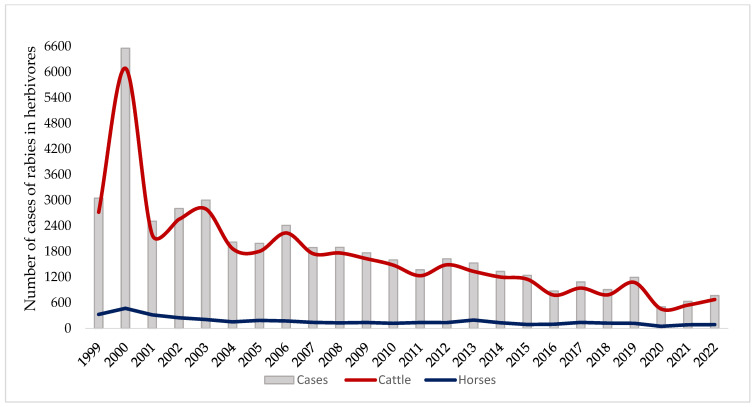
Annual distribution of the number of rabies cases in herbivores in Brazil (1999–2022). Source: Brazil, Ministry of Agriculture, Livestock, and Supply, Animal Health Information System, Brasília, DF: MAPA, 2023 [23].

Brazil is the fifth largest country in terms of territorial extension and plays an important role in livestock production at both the national and international levels, boasting the largest herds in Latin America. However, livestock growth is intricately linked to environmental challenges, particularly escalating deforestation, which primarily takes place in the Amazon region of Brazil and leads to substantial landscape modification [19]. The absence of a consistent strategic plan for agricultural and livestock expansion not only irreversibly alters the landscape but also increases the risk of infectious diseases, notably rabies, in humans and other mammals owing to the adaptability of hematophagous bats to environmental changes [20]. Rabies in herbivores is endemic throughout Brazil, with higher incidences in the states of Tocantins, Goiás, Rio Grande do Sul, Minas Gerais, and São Paulo, which have historically reported more cases of rabies in cattle due to the more significant presence of reservoirs such as vampire bats. From 1999 to December 2022, 50,944 cases of rabies in herbivores were recorded nationwide (Figure 1). In 2021, 661 cases were reported, with São Paulo leading (17%), followed by Minas Gerais (14.3%), and Paraná (10.1%). In 2022, 776 cases were registered, of which 677 affected cattle [21].

The annual temporal analysis of bovine rabies cases from 1999 to 2022 revealed notable peaks in 2000 (6088 cases) and 2003 (2795 cases), with significant fluctuations until 2006. Subsequently, a gradual decrease occurred with minor oscillations until 2019, with a notable reduction observed in the subsequent years, reaching the lowest reported case values. Regarding rabies cases in horses, the blue timeline displayed slight fluctuations throughout the examined period, with a peak in 2000 that totaled 470 cases (Figure 1). Despite governmental efforts to establish guidelines for herbivore rabies control, current evidence consistently points to a global trend of underreporting rabies cases. This underreporting can compromise the accuracy of analyses and the comprehensive understanding of the actual incidence of rabies in herbivores, which is influenced by factors such as surveillance system limitations and insufficient resources for precise diagnostics [22].

### 1.3. Impacts on Human Health

More than 99% of rabies cases in herbivores in Brazil are caused by hematophagous bats of the species *Desmodus rotundus*. The ability of these bats to cover distances exceeding 10 km and utilize various types of forests, especially riparian zones, to traverse extensive agricultural areas allows the virus to spread from an infected animal across state and national borders. The adaptability of *D. rotundus* to artificial shelters and its proximity to cattle on farms and rural populations influences rabies outbreaks in both cattle and humans, thus contributing to disease transmission in agricultural environments. The preference of these bats for artificial shelters highlights the need for specific control measures to mitigate risks associated with virus transmission [23,24].

Human rabies, although rare in Brazil, remains a relevant concern, with an average of seven cases per year over the last ten years. Notably, a recent epidemiological shift has been observed, where the antigenic variant AgV-3, associated with hematophagous bats, has been detected in the majority of human rabies cases. In 2017, of the six registered rabies cases, none were related to dog bites, whereas five cases were attributed to bats, and one to a feline bite. In 2018, all 11 cases were attributed to bats, and in 2019, the only reported case was caused by a feline bite from an animal infected with an antigenic variant of bat AgV-3. In 2022, five confirmed rabies cases were associated with the antigenic variant of the bat. Additionally, in 2023, one human rabies case was linked to the transmission of bovine rabies [25].

**Figure 2:**
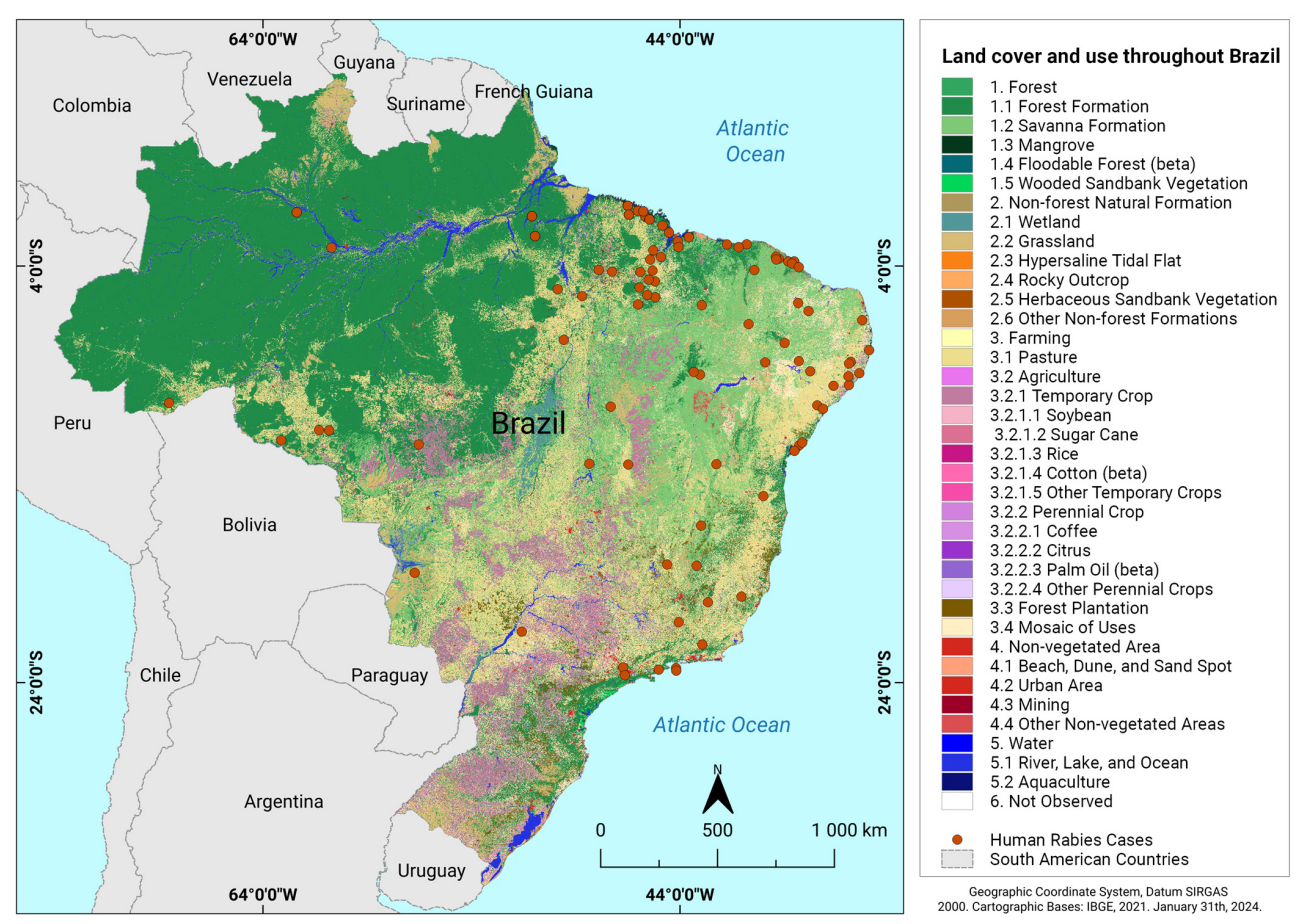
Relationship between the number of human rabies cases and changes in vegetation coverage and land use in Brazilian regions.

In this context, bats play an increasingly significant role in the spread of rabies, with the periodic movement of their colonies becoming more pronounced [26]. This increase in bat movement is related to the deforestation that has resulted from urban expansion and the conversion of natural ecosystems into areas for intensive livestock farming. Notably, there has been a loss of natural habitats, such as forests and riparian areas, for these animals, which reduces the availability of shelter, reproduction sites, and food [27]. These alterations have a direct impact on the dynamics of bat communities in various regions of Brazil and leads animals to seek more favorable locations that offer better shelter and food conditions. This behavior also results in a closer proximity to humans and an increased susceptibility of communities to rabies [28]. Figure 2 complements this dynamic by illustrating the corresponding increase in the number of human rabies cases in regions with significant alterations in vegetation cover and land use. The direct relationship among bat behavior, environmental changes, and the increase in rabies cases is visually evident, which emphasizes the need for improving our understanding and providing effective intervention for rabies in these areas.

### 1.4. Mitigating the Impact of Rabies

Efforts to mitigate the impact of bovine rabies in Brazil include vaccination campaigns, public awareness programs, and improvements in surveillance and reporting systems. The vaccination of animals, particularly herbivores like cattle, is essential for controlling the disease, especially in endemic areas, and is conducted according to the guidelines of the Ministry of Agriculture, Livestock, and Supply (MAPA). MAPA organizes annual vaccination campaigns in high-risk areas, particularly those prone to outbreaks caused by vampire bats. In some regions, vaccination is mandatory, and farmers must vaccinate their animals and maintain vaccination records [18].

Control measures also include habitat management and population control of vampire bats using anticoagulant pastes. Additionally, educational programs are implemented to inform farmers about the importance of vaccination and best practices for rabies prevention. Despite these efforts, herbivorous rabies remains a concern, highlighting the need for ongoing surveillance and control measures [18,23].

The persistence of herbivorous rabies in Brazil presents significant challenges to public health and has a substantial economic impact. In addition to losses in the livestock industry, control measures such as quarantine and surveillance impose considerable financial burdens on farmers and governments. Herbivorous rabies also reduces the productivity of affected cattle and affects operational efficiency and sector profitability [23].

Additionally, herbivorous rabies outbreaks, especially in cattle, can cause significant financial losses due to blood spoliation. This phenomenon not only reduces animal productivity but also leads to increased veterinary care and management costs, highlighting public health risks due to the zoonotic nature of the disease. The potential transmission to humans underscores the urgency for substantial investments in vaccination campaigns, surveillance, and control, which imposes a significant financial burden on governments and cattle producers [29]. A thorough understanding of these complexities reinforces the need for integrated approaches and ongoing investments to mitigate the impact of bovine rabies on Brazil.

Vaccinating bats is not viable, as such vaccination would require passive and/or active search campaigns. Given the wide geographical distribution of these animals, this task would be impractical. It would necessitate the monitoring and counting of nests, roosts, and bat assemblies, information that is currently unavailable.

## 2. Conclusions

Rabies represents a complex and multifaceted challenge in Brazil, where the disease significantly affects public health and the livestock industry. Virus transmission, primarily by hematophagous bats, underscores the urgency of integrated strategies to control its spread. Vaccination is a crucial strategy for rabies control in herbivores and plays a central role in safeguarding livestock to ensure food security and prevent rabies transmission to humans. The interaction between deforestation and the bat movement emphasizes the importance of environmental approaches to mitigate the threat of rabies. Beyond the economic impact on the livestock industry, the risks to human health underscore the need for substantial investment in awareness campaigns and research. The complexity of this scenario requires collaboration among health authorities, researchers, and agricultural communities to implement comprehensive preventive measures. The pursuit of innovative and sustainable solutions is essential to ensure the safe coexistence of animals and humans and to effectively mitigate the associated risks of rabies in Brazil.

## Data Availability

The original contributions presented in the study are included in the article, further inquiries can be directed to the corresponding authors.

## References

[1] World Health Organization, Food and Agriculture Organization of the United Nations (FAO), World Organisation for Animal Health (OIE) (2018). Zero by 30: The Global Strategic Plan to End Human Deaths from Dog-Mediated Rabies by 2030.

[2] Conway D.J., Roper C. (2000). Micro-evolution and emergence of pathogens. Int. J. Parasitol..

[3] World Health Organization Rabies. https://www.who.int/news-room/fact-sheets/detail/rabies.

[4] World Health Organization PEP Recommendations. https://www.who.int/teams/control-of-neglected-tropical-diseases/rabies/pep-recommendations.

[5] Ministério da Saúde: Brasil, Secretaria de Vigilância em Saúde (2022). Coordenação Geral de Vigilância de Zoonoses e Doenças de Transmissão Vetorial. Guia de Vigilância em Saúde.

[6] Sodré D.N.A., Rossi G.A.M., Mathias L.A., de Andrade Belo M.A. (2023). Epidemiology and Control of Rabies in Cattle and Equines in Rondônia State, a Brazilian’s Legal Amazon Area. Animals.

[7] Gomes M.N., Monteiro A.M., Lewis N., Gonçalves C.A., Nogueira Filho V.S. (2007). Áreas propícias para o ataque de morcegos hematófagos Desmodus rotundus em bovinos na região de São João da Boa Vista, Estado de São Paulo. Pesqui. Vet. Bras..

[8] Chomel B.B., Sykes J.E., Sykes J.E. (2021). 21-Rabies. Infectious Diseases of the Dog and Cat.

[9] Gomes M.N., Monteiro A.M.V., Escada M.I.S. (2011). Raiva bovina segundo os mosaicos de uso e cobertura da terra no estado de São Paulo entre 1992 e 2003. Arq. Bras. Med. Vet. Zootec..

[10] Horta M.A., Ledesma L.A., Moura W.C., Lemos E.R.S. (2022). From dogs to bats: Concerns regarding vampire bat-borne rabies in Brazil. PLoS Negl. Trop. Dis..

[11] Santos A.J.F., Ferreira J.M., Baptista F., Alexandrino B., Silva M.A.G., Gomes J.E.C., Júnior J.P.V., Tavares R.M., de Sousa Almeida K. (2022). Statistical analysis between 2006 and 2019 and forecast of rabies in cattle from 2020 to 2022 in Tocantins State (Brazil), by using the R Studio software. Epidemiol. Infect..

[12] Duarte N.F.H., Alencar C.H., Cavalcante K.K.S., Correia F.G.S., Romijn P.C., Araujo D.B., Favoretto S.R., Heukelbach J. (2020). Increased detection of rabies virus in bats in Ceará State (Northeast Brazil) after implementation of a passive surveillance programme. Zoonoses Public Health.

[13] Wada M.Y., Rocha S.M., Maia-Elkhoury A.N.S. (2011). Situação da raiva no Brasil, 2000 a 2009. Epidemiol. Serv. Saude.

[14] Lima E.F., Riet-Correa F., Castro R.S., Gomes A.B., Lima F.S. (2005). Sinais clínicos, distribuição das lesões no sistema nervoso e epidemiologia da raiva em herbívoros na região Nordeste do Brasil. Pesqui. Vet. Bras..

[15] Yao Y., Zhang Z., Yang Z. (2023). The combination of vaccines and adjuvants to prevent the occurrence of high incidence of infectious diseases in bovine. Front. Vet. Sci..

[16] Almeida M.F., Queiroz L.H. (2023). History of Rabies in Brazil.

[17] Voigt C.C., Voigt-Heucke S.L., Schneeberger K. (2012). Isotopic Data Do Not Support Food Sharing within Large Networks of Female Vampire Bats (*Desmodus rotundus*). Ethology.

[18] (2009). Brasil, Ministério da Agricultura, Pecuária e Abastecimento, Secretaria de Defesa Agropecuária. Controle da Raiva de Herbívoros no Brasil. https://www.gov.br/agricultura/pt-br/assuntos/sanidade-animal-e-vegetal/saude-animal/programas-de-saude-animal/raiva-dos-herbivoros-e-eeb/copy2_of_MANUAL_RAIVAHERBVOROS2009.pdf.

[19] Benavides J.A., Megid J., Campos A., Hampson K. (2020). Using Surveillance of Animal Bite Patients to Decipher Potential Risks of Rabies Exposure From Domestic Animals and Wildlife in Brazil. Front. Public Health.

[20] Kobayashi Y., Sato G., Mochizuki N., Hirano S., Itou T., Carvalho A.A., Albas A., Santos H.P., Ito F.H., Sakai T. (2008). Molecular and geographic analyses of vampire bat-transmitted cattle rabies in central Brazil. BMC Vet. Res..

[21] Mello A.K.M., Brumatti R.C., Neves D.A., Alcântara L.O., Araújo F.S., Gaspar A.O., Lemos R.A. (2019). Bovine rabies: Economic loss and its mitigation through antirabies vaccination. Pesqui. Vet. Bras..

[22] Oliveira F.A.S., Castro R.J.S., de Oliveira J.F., Barreto F.M., Farias M.P.O., Marinho G.L.O.C., Soares M.J.D.S., Silva-Júnior A., Schwarz D.G.G. (2022). Geographical and temporal spread of equine rabies in Brazil. Acta Trop..

[23] Animal Health Information System Ministry of Agriculture, Livestock and Supply. https://www.gov.br/agricultura/pt-br/assuntos/sanidade-animal-e-vegetal/saude-animal/epidemiologia/portugues.

[24] Mantovan K.B., Menozzi B.D., Paiz L.M., Sevá A.P., Brandão P.E., Langoni H. (2022). Geographic Distribution of Common Vampire Bat Desmodus rotundus (Chiroptera: Phyllostomidae) Shelters: Implications for the Spread of Rabies Virus to Cattle in Southeastern Brazil. Pathogens.

[25] Ministério da Saúde: Brasil, Secretaria de Vigilância em Saúde Casos de Raiva Humana Segundo Especie de Animal Agressor, 1986–2023. https://www.gov.br/saude/pt-br/assuntos/saude-de-a-a-z/r/raiva/imagens/arquivos-2023/atualizacoes-16-05-2023/casos-de-raiva-humana-segundo-especie-de-animal-agressor-1986-2023-brasil/view.

[26] Orlando A., Panchana V.F., Caldero J.L., Muñoz O.S., Campos D.N., Torres-Lasso P.R., Arcos F.J., Quentin E. (2019). Risk Factors Associated with Attacks of Hematophagous Bats (Desmodus rotundus) on Cattle in Ecuador. Vector Borne Zoonotic Dis..

[27] Ribeiro J., Staudacher C., Martins C.M., Ullmann L.S., Ferreira F., Araujo J.P., Biondo A.W. (2018). Bat rabies surveillance and risk factors for rabies spillover in an urban area of Southern Brazil. BMC Vet. Res..

[28] Ulloa-Stanojlovic F.M., Dias R.A. (2020). Spatio-temporal description of bovine rabies cases in Peru, 2003–2017. Transbound. Emerg. Dis..

[29] Santos B.L., Bruhn F.R.P., Coelho A.C.B., Estima-Silva P., Echenique J.V., Sallis E.S.V., Schild A.L. (2019). Epidemiological study of rabies in cattle in southern Brazil: Spatial and temporal distribution from 2008 to 2017. Pesqui. Vet. Bras..

